# Population Pharmacokinetics and Limited Sampling Strategy for Therapeutic Drug Monitoring of Polymyxin B in Chinese Patients With Multidrug-Resistant Gram-Negative Bacterial Infections

**DOI:** 10.3389/fphar.2020.00829

**Published:** 2020-06-05

**Authors:** Peile Wang, Qiwen Zhang, Zhenfeng Zhu, Min Feng, Tongwen Sun, Jing Yang, Xiaojian Zhang

**Affiliations:** ^1^Department of Pharmacy, First Affiliated Hospital of Zhengzhou University, Zhengzhou, China; ^2^Henan Key Laboratory of Precision Clinical Pharmacy, Zhengzhou University, Zhengzhou, China; ^3^Department of ICU, First Affiliated Hospital of Zhengzhou University, Zhengzhou, China; ^4^Department of General ICU, First Affiliated Hospital of Zhengzhou University, Zhengzhou, China

**Keywords:** polymyxin B, population pharmacokinetics, limited sampling strategy, therapeutic drug monitoring, multidrug-resistant Gram-negative bacterial infection

## Abstract

Polymyxin B is used as a last therapeutic option for the treatment of multidrug-resistant Gram-negative bacterial infections. This study aimed to develop a population pharmacokinetic model and limited sampling strategy, a method to estimate the area under the concentration curve (AUC) by using a limited number of samples, to assist therapeutic drug monitoring of polymyxin B in Chinese patients. Population pharmacokinetic analysis was performed using Phoenix^®^ NLME with data obtained from 46 adult patients at steady state. Various demographic variables were investigated as potential covariates for population pharmacokinetic modeling. The limited sampling strategies based on the Bayesian approach and multiple linear regression were validated using the intraclass correlation coefficient and Bland-Altman analysis. As a result, the data was described by a two-compartment population pharmacokinetic model. Through the modeling, creatinine clearance was found to be a statistically significant covariate influencing polymyxin B clearance. The limited sampling strategies showed the two-point model (C_0h_ and C_2h_) could predict polymyxin B exposure with good linear relativity (r^2^ > 0.98), and the four-point model (C_1h_, C1_.5h_, C_4h_, and C_8h_) performed best in predicting polymyxin B AUC (r^2^ > 0.99). In conclusion, this study successfully developed a population pharmacokinetic model and limited sampling strategies that could be applied in clinical practice to assist in therapeutic drug monitoring of polymyxin B in Chinese patients.

## Introduction

Polymyxin B, a polypeptide antibiotic obtained from the fermentation products of *Paenibacillus polymyxa*, was first used clinically in the 1950s but was largely abandoned in the 1970s due to nephrotoxicity and neurotoxicity. Recently, with the prevalence of multidrug-resistance (MDR) Gram-negative bacterial infections, polymyxin B has been re-introduced to clinical practice against these challenging infections. It is currently considered as the last drug resort for MDR Gram-negative bacterial infections (Velkov et al., 2013; Nation et al., 2015).

Despite being used clinically for decades, few pharmacokinetic/pharmacodynamics (PK/PD) data is available for polymyxin B (Tam et al., 2005; Bergen et al., 2012; Tsuji et al., 2016; Lakota et al., 2018; Landersdorfer et al., 2018). These studies found the area under the concentration-time curve to minimum inhibitory concentration ratio (fAUC: MIC), as the PK/PD index, appeared to be a good efficacy predictor for polymyxin B. Accordingly, a global multidisciplinary panel of infectious diseases and pharmacotherapy experts recommended therapeutic drug monitoring (TDM) was required to optimize the clinical use of polymyxin B. An AUC across 24 hours at steady state (AUC_ss,24_ _hr_) target of 50 to 100 mgċh/L, corresponding to a C_ss,avg_ of 2 to 4 mg/L, might be acceptable for polymyxin B therapy (Tsuji et al., 2019). However, the guidelines also noted that data were lacking for PK properties and AUC_ss,24_ _hr_ targets of polymyxin B, and the recommended therapeutic target largely based on *in vitro* and animal data rather than clinical outcome (Alosaimy et al., 2019). In China, polymyxin B is only commercially available since 2017, there is almost no PK data on polymyxin B from Chinese patients. Therefore, it is necessary to assess the population PK of polymyxin B in Chinese patients.

In addition, to calculate AUC, it requires multiple blood draws during a dosing interval to get a full pharmacokinetic curve. This is not only time-consuming and expensive but also a huge burden to patients, and therefore unfeasible in clinical practice. To solve this problem, the limited sampling strategy (LSS), estimating AUC with one or a few samples, would be suitable in the clinic (David and Johnston, 2000). It has been proposed for TDM of many drugs, such as mycophenolate mofetil and colistin (Zhang et al., 2018; Kim et al., 2019; Van Der Galiën et al., 2020). However, no LSS report is available for polymyxin B at present.

Therefore, this study aimed to develop and validate a population PK model, along with clinically feasible LSS for TDM of polymyxin B in Chinese patients with MDR Gram-negative bacterial infections.

## Materials and Methods

### Patients Demographics

A single-center clinical trial was conducted between April 2018 and November 2019 at the First Affiliated Hospital of Zhengzhou University. The study protocol was approved by the hospital ethics committee review board, and all participating subjects signed the informed consent (Zhengzhou University Medical Research and Ethics Committee, No. L2018K129).

Patients who were ≥ 18 years of age, received intravenous polymyxin B (sulfate; polymyxin B injection, Shanghai First Biochemical Pharmaceutical Co., Ltd., China) for ≥ 72 hours, and were documented MDR Gram-negative bacterial infections were included. Patients with renal replacement therapy were excluded. Demographic characteristics (age, sex, and body weight), polymyxin B therapy, and laboratory data before polymyxin B TDM were collected from electronic medical records, including alanine aminotransferase (ALT), aspartate aminotransferase (AST), glutamyl transpeptidase (GGT), alkaline phosphatase (ALP), urea nitrogen, serum creatinine (Scr), serum uric acid, serum proteins, serum albumin, total bilirubin (TBIL), direct bilirubin (DBIL), and creatinine clearance (CrCL). CrCL was calculated using the Cockcroft-Gault equation with body weight (Janmahasatian et al., 2005).

### Polymyxin B Administration and Sample Collection

According to the Chinese package insert, the maintenance dose of polymyxin B was 50 to 100 million units (1 million units equal to 1 mg) twice daily. The loading dose was 100 to 150 million units in clinical practice. The recommended infusion time was one hour but could be lengthened as needed. Polymyxin B treatment including dose, infusion time, and duration of therapy depended on the medical teams.

Blood samples were obtained after 3 days of therapy. On day 4, one blood sample (2 ml, C_0h_) was collected immediately at pre-dose, and five to seven blood samples (mainly C_0.5h_, C_1h_, C_1.5h_, C_2h_, C_4h_, C_6h_, and C_8h_) were collected at pre-next dose into EDTA tubes for each patient. To calculate AUC_0–12h_, C_0h_ was regarded as C_12h_ for those who hid not obtained C_0h_. All samples were centrifuged at 3,500×*g* for 10 min. The supernatant was collected and stored at −80°C until analysis.

### Quantification of Polymyxin B Concentrations

Plasma concentrations of polymyxin B were determined using liquid chromatography-tandem mass spectrometry (LC-MS/MS) in the hospital laboratory. The method was previously described by our group (Wang et al., 2020). In brief, the calibration curves showed acceptable linearity over 0.2 to 10 µg/ml for polymyxin B1and 0.05 to 2.5 µg/ml for polymyxin B2. The upper limit of quantification was extended to 20 µg/ml for polymyxin B1 and 5.0 µg/ml for polymyxin B2 after four-fold dilution. For precision and accuracy, the intra- and inter-day imprecision of polymyxin B1 and B2 was less than 13.93%, and the inaccuracy ranged from −10.87 to 11.13%. Since polymyxin B1 and B2 had similar structures, molecular weight, pharmacological activities, and pharmacokinetic characteristics, the plasma concentration of polymyxin B was summed to derive the total polymyxin B1 and B2 concentrations (Tam et al., 2011; Manchandani et al., 2016a).

### Population Pharmacokinetics Analysis

Based on a previously published study (Avedissian et al., 2019), the PK parameters were determined by one- and two-compartmental models performed using Phoenix^®^ NLME software (v7.0, Pharsight, Mountain View, CA, USA). Initial PK parameters were estimated by the Naive-pooled model. PK models were estimated by the first-order conditional

(1) $$P_{i}=\theta \times \exp(\eta_{i})$$

where *P*_i_ is the PK parameters of the i^th^ patient, *θ* is the population PK pharmacokinetic parameters, and *η*_i_ is a normally distributed random variable with a mean of 0 and a variance of *ω*^2^. Intra-individual variability (residual error) is described using additive (C_obs_ = C_pred_ + *ϵ*), proportional [C_obs_ = C_pred_ × (1+ *ϵ*)] or mixed (additive + proportional) models, where C_obs_ and C_pred_ are the observed and predicted concentrations, and *ϵ* is an error variable with a mean of 0 and a variance of *σ*^2^. Model assessment criteria included precision of parameter estimates (standard error), goodness-of-fit plots, and likelihood ratio test (−2LL). For the one-compartment model, basic PK parameters were the volume of central compartment distribution (V) and central compartment clearance (Cl). For the two-compartment model, there were two additional parameters as the volume of peripheral compartment distribution (V2) and inter-compartmental clearance (Q).

In the process of model development, age, sex, body weight, ALT, AST, GGT, ALP, urea nitrogen, Scr, serum uric acid, serum proteins, serum albumin, TBIL, DBIL, and CrCL were evaluated as the covariates. The covariates selection was evaluated using a stepwise process (a forward-selection process and then a backward-elimination process). By comparing with initial model, the inclusion criteria for covariates was a drop > 3.84 (*P* = 0.05) of objective function (OFV; −2LL) during forward-selection. Then each selected covariate was re-evaluated by backward-elimination. An increase of OFV > 6.63 (*P* = 0.01) was required for confirmation.

After covariate selection, the correlations between population PK parameters were graphically examined in the scatter plot. The relevant parameters were introduced to non-diagonal random effects. A drop of OFV > 6.63 (*P* = 0.01) was retained to obtain the final model.

Model validation was assessed by using plots of observed concentrations (DV) versus population predicted concentrations (PRED) or individual predicted concentrations (IPRED), and conditional weighted residuals (CWRES) versus time (IVAR) or PRED. Additionally, the model performance was evaluated by a prediction-corrected visual predictive check (VPC). The 5^th^, 50^th^, and 95^th^ quantiles and the 90% confidence intervals (CIs) of 5^th^, 50^th^, and 95^th^ quantiles (200 replicates) were developed from the final model parameters. The model stability was assessed using bootstrap analysis (1,000 bootstrap samples). The median, standard error (SE), and coefficient of variation (CV%) were calculated from the empirical bootstrap distribution and compared estimates with the original dataset.

### Model-Based Simulation

For optimal dose selection, the plasma concentration-time profile of 1,000 individuals was simulated using the final population PK model. The following dose regimens were evaluated: 100 mg loading dose with 50 mg maintenance dose twice daily, 150 mg loading dose with 75 mg maintenance dose twice daily, and 150 mg loading dose with 100 mg maintenance dose twice daily. The infusion rate was set as 50 mg/h. The covariate of CrCL was selected as 31.3, 105.9, and 315.2 ml/min. which were the low, medium, and high values from the sample of patients.

### Limited Sampling Strategies

The AUC_0-12h_ of polymyxin B was calculated by the linear-up-log-down method. LSSs were investigated with Bayesian analysis and linear regression analysis. By using the Bayesian approach, Polymyxin B predicted concentrations were estimated from limited point values with the final population PK model. The observed and predicted AUC_0-12h_ were analyzed using the Pearson correlation coefficient (r). The linear regression analysis was performed on IBM SPSS Statistics (v24.0, SPSS Inc., Chicago, USA). The stepwise forward multiple regression method was used to investigate the correlation between AUC_0-12h_ and polymyxin B concentrations at different time points. The determination coefficient (r^2^) evaluated the regression level of the equation. Good regression equations were selected for model validation.

The model was internally validated by the Jackknife method. One sample was removed from the original sample at a time, then regression analysis of the remaining sample was performed to obtain a new equation and calculate the AUC_0-12h_ of the removed one sample. Prediction error (PE, Equ. 2) and root mean square error (RMSE, Equ. 3) were used to evaluate the accuracy and precision of the model. The acceptance criteria of RMSE < 15 and r^2^ > 0.95 (Van den Elsen et al., 2018).

(2) $$\text{PE}(\%)=({\text{AUC}}_{\text{predicted}}-{\text{AUC}}_{\text{measured}})/{\text{AUC}}_{\text{measured}}\;\times 100\%$$

(3) $$\text{RMSE}=\sqrt{1/n\Sigma {\left(\text{PE}\%\right)}^{2}}$$

The consistency between the measured and predicted value of AUC_0-12h_ was evaluated by intraclass correlation coefficient (ICC), and Bland-Altman (BA) analysis. The BA analysis was expressed by the scatter diagram and limits of agreement. The lower limit of 95% CI < 0.9 and the limits of agreement within ±15% were acceptable for the clinic (Zhang et al., 2018). The best model was selected based on the values of r^2^, PE, RMSE, ICC, and BA analysis.

## Results

### Patients

A total of 46 patients contributed to 331 plasma samples were enrolled in this study. The patients' demographic and clinical information were listed in **Table 1**. Among them, pathogenic bacteria cultures results showed that sputum was the most common primary site of infection, along with blood, cerebrospinal fluid, and puncture fluid, etc. MDR *Acinetobacter baumannii* and *Klebsiella pneumoniae* were the major causative agent of the infections, and 6 patients were infected with two kinds of MDR Gram-negative bacterial.

**Table 1:** Demographic characteristics of patients.

Characteristics	Values (n = 46)
Gender	
Male, %	39 (84.78%)
Female, %	7 (15.22%)
Age (year)	46 (18–94)
Weight (kg)	70 (45–98)
Creatinine clearance (mlċmin^−1^)	89.3 (15.6–315.2)
Serum creatinine (µmolċL^−1^)	73.0 (21.0–387.0)
Urea nitrogen (mmolċL^−1^)	9.8 (2.4–59.3)
Uric acid (µmolċL^−1^)	170.5 (19.0–600.0)
Alanine aminotransferase (UċL^-1^)	31.0 (3.0–336.0)
Aspartate aminotransferase (UċL^−1^)	40.0 (12.0–206.0)
Glutamyl transpeptidase (UċL^−1^)	59.5 (2.0–663.0)
Alkaline phosphatase (UċL^−1^)	122.0 (44.0–334.0)
Total protein (gċL^−1^)	57.2 (42.7–75.3)
Serum albumin (gċL^−1^)	31.5 (18.3–43.6)
Total bilirubin (µmolċL^−1^)	16.1 (3.6–286.4)
Direct bilirubin (µmolċL^−1^)	9.4 (1.6–228.9)
Daily dose/body weight (mg·kg^−1^)	1.91 (1.18–3.33)
Daily dose	
100 mg, %	25 (50.0%)
150 mg, %	15 (32.6%)
200 mg, %	8 (17.4%)
Injection duration	
0.5 h, %	2 (4.35%)
1 h, %	37 (80.43%)
2 h, %	7 (15.22%)
Pathogenic bacteria cultures	
*Acinetobacter baumannii*	19
*Klebsiella pneumoniae*	20
*Pseudomonas aeruginosa*	7
*Escherichia coli*	4
Others	2

Values are median (range) or No. (%).

### Population PK Model

Preliminary analysis of base model showed the OFVs of one- and two-compartmental models were 1062.13 and 694.63, respectively. Based on OFV, CV values, and diagnostic scatter plots, a two-compartment model with a proportional option was chosen as the base model. In the next step, covariate model building identified CrCL as the covariate for Cl (ΔOFV = 8.26, *P* < 0.01). Age, gender, and other laboratory data had no significant effect on population PK parameters. Furthermore, a strong correlation between Cl, Cl2, and V was observed, and then incorporated it into non-diagonal random effects (ΔOFV = 30.60, *P* < 0.01). The correlations between V-Cl, V-V2, and Cl-V2 were 0.713, 0.667, and 0.571, respectively. Accordingly, the final PK model was shown in Equ. 4 to 7, where 105.9 ml/min was the median of CrCL.

(4) V(L)=6.218×exp(ηV)

(5) V2(L)=11.922×exp(ηV2)

(6) $$\text{Cl}(\text{L}/\text{h})=1.786{\left(\frac{\text{CrCl}}{105.9}\right)}^{0.362}\times \text{exp}(\eta \text{Cl})$$

(7) Q(L/h)=13.518×exp(ηQ)

**Figure 1:**
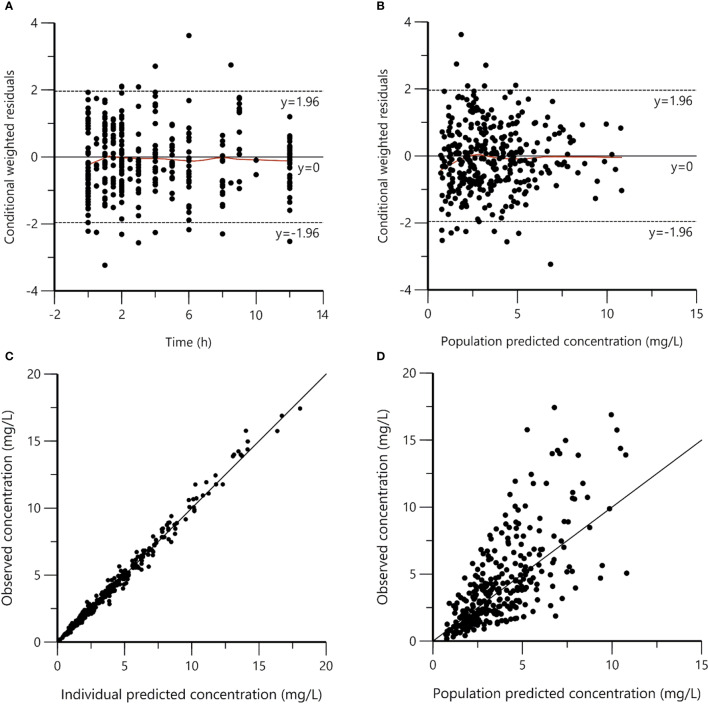
Goodness-of-fit plots for the final population pharmacokinetic model. **(A)** Conditional weighted residuals versus time (CWRES vs. IVAR); **(B)** Conditional weighted residuals versus population predicted concentrations (CWRES vs. PRED); **(C)** Observed versus individual predicted concentrations (DV vs. IPRED); **(D)** Observed versus population predicted concentrations (DV vs. PRED). The reds lines in panels **(A, B)** represent smoothed regression lines.

**Figure 2:**
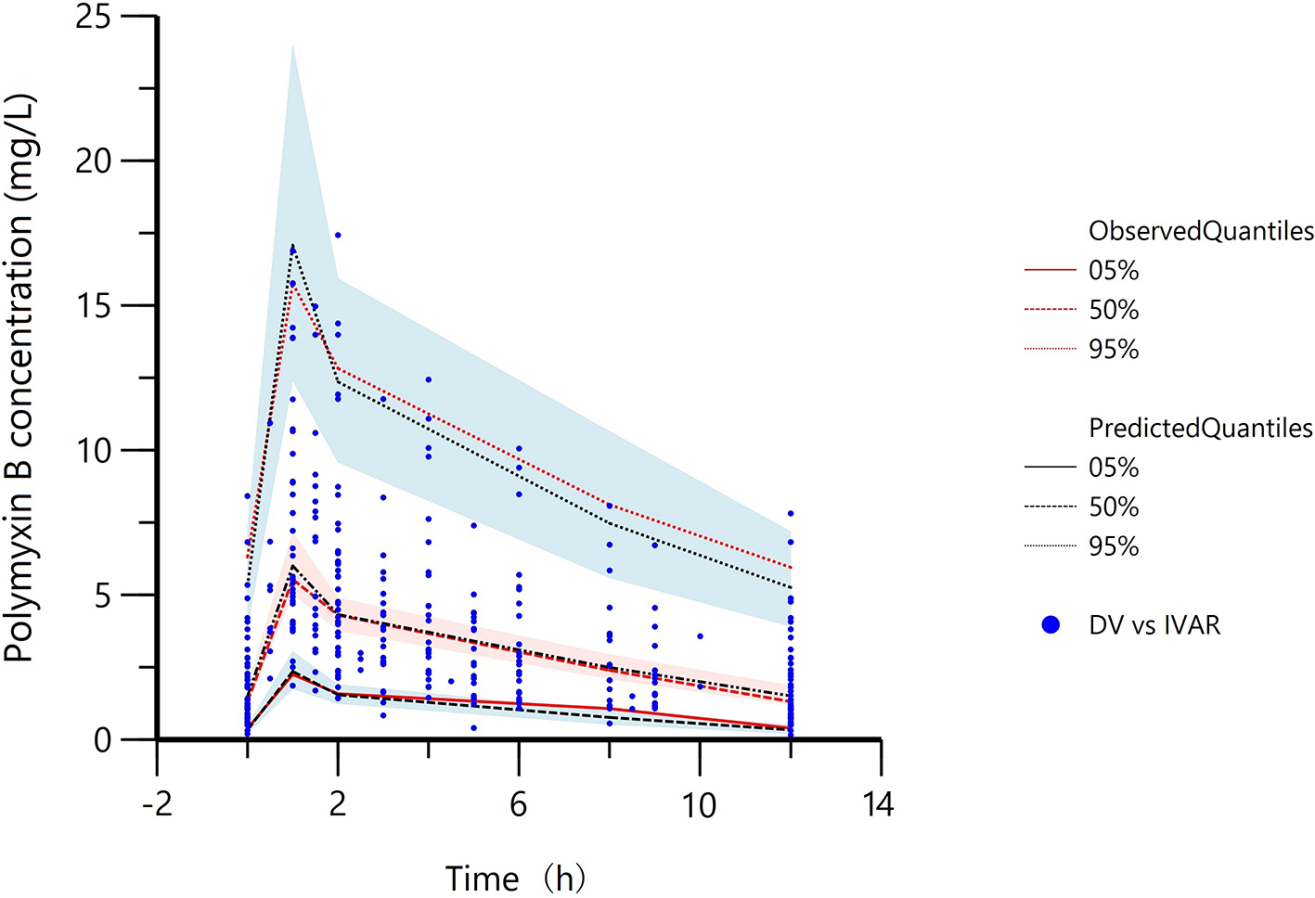
Prediction corrected-visual predictive check of the final model. Red lines represent the 5^th^, 50^th^, and 95^th^ percentiles of the observed concentrations; the shaded areas represent the 90% confidence intervals of the 5^th^, 50^th^, and 95^th^ percentiles of the simulated concentrations, respectively; the dots represent the observed data; DV, observed concentration; IVAR, Time.

The goodness-of-fit plots for the final model were shown in **Figure 1**. The observed concentrations were consistent with PRED and IPRED, and the plots of CWRES vs time and PRED were normally distributed. The estimated covariates and bootstrap replicates were shown in **Table 2**, which indicated the final model had qualified stability. In prediction corrected-VPC (**Figure 2**), most of the observed plots were distributed within the 90% CIs of predicted corresponding quantiles, which indicated the prediction of simulated data matched the observed plots.

**Table 2:** Parameter estimates and bootstrap results of the final population pharmacokinetic model.

Parameter	Final model				Bootstrap			
	Estimate	SE	CV (%)	Shrinkage (%)	Median	SE	CV(%)	95% CI
tvV	6.218	0.83	13.33	12.31	5.960	0.92	15.50	4.169–8.090
tvV2	11.922	1.74	14.62	0.66	12.073	1.71	14.17	8.705–15.960
tvCl	1.786	0.12	6.75	8.45	1.771	0.09	5.18	1.581–1.964
tvQ	13.518	3.35	24.82	17.57	14.427	3.68	25.50	8.157–23.928
dCldCrCL	0.362	0.09	24.82	NA	0.357	0.07	20.47	0.196–.513
Inter-individual variability								
*ω*^2^V	0.318	0.14	43.71	NA	0.354	0.14	39.27	NA
*ω*^2^Cl	0.208	0.04	21.15	NA	0.204	0.03	16.18	NA
*ω*^2^V2	0.690	0.20	29.42	NA	0.660	0.19	28.94	NA
*ω*^2^Q	1.508	0.46	30.44	NA	1.458	0.45	31.48	NA
CorrV-Cl	0.713	0.06	8.13	NA	0.681	0.08	11.31	NA
CorrV-V2	0.667	0.13	19.49	NA	0.630	0.12	19.84	NA
CorrCl-V2	0.571	0.07	12.26	NA	0.578	0.06	10.03	NA
Residual variability (*σ*)								
stdev0	0.110	0.01	5.30	NA	0.110	0.01	7.13	0.093–0.127

SE, standard error; CV%, percent confidence of variation; CI, confidence interval; tvV, typical value of volume of central compartment distribution (V); tvV2, typical value of volume of peripheral compartment distribution (V2); tvCl, typical value of central compartment clearance (Cl); tvQ, typical value of inter-compartmental clearance (Q, Cl2); dCldCrCL, fixed parameter coefficient of creatinine clearance (CrCL) to Cl; ωV, variance of inter-individual variability for V; CorrV-Cl, correlation between V and Cl; stdev0, standard deviation; NA, not applicable.

### Model-Based Simulation

**Figure 3:**
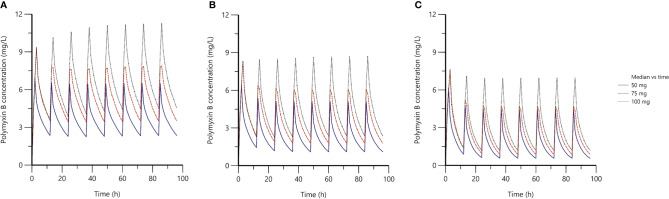
The median simulated plasma concentration-time profiles based on the final population PK model. **(A)** the creatinine clearance (CrCL) of 31.3 ml/min; **(B)** the CrCL of 105.9 ml/min; **(C)** the CrCL of 315.2 ml/min; the blue solid lines represented 100 mg loading dose with 50 mg maintenance dose twice daily; the red dash solid lines represented 150 mg loading dose with 75 mg maintenance dose twice daily; the black dot lines represented 150 mg loading dose with 100 mg maintenance dose twice daily.

The median simulated plasma concentration-time profiles of polymyxin B based on different doses and CrCL values were displayed in **Figure 3**, and the simulated AUC_24h_ and C_ss,avg_ of polymyxin B on day four was quantified in **Table 3**.

**Table 3:** The simulated AUC_24h_ of polymyxin B on day four based on the final population pharmacokinetic model.

Maintenance dose	CrCL (ml/min)	AUC_24h_ (mg·h/L)			C_ss,avg_ (mg/L)		
		P5	P50	P95	P5	P50	P95
50 mg, q12h	31.3	36.93	87.20	192.19	1.54	3.63	8.01
	105.9	23.85	53.33	118.77	0.99	2.22	4.95
	315.2	15.54	37.92	84.49	0.65	1.58	3.52
75 mg, q12h	31.3	56.60	128.58	295.12	2.36	5.36	12.30
	105.9	35.15	82.04	180.81	1.46	3.42	7.53
	315.2	22.46	53.60	121.32	0.94	2.23	5.06
100 mg, q12h	31.3	75.39	167.90	364.58	3.14	7.00	15.19
	105.9	49.23	108.57	247.01	2.05	4.52	10.29
	315.2	30.29	71.91	167.40	1.26	3.00	6.98

AUC24h, area under the plasma concentration-time curve over 24 hours; P5, 5^th^ percentile; P50, 50^th^ percentile; P95, 95^th^ percentile; q12h, every 12 hours.

### Limited Sampling Strategy

The AUC_0-12__h_ of 46 patients was 43.64 ± 27.68 mg·h/L with a range of 8.50 to 122.84 mg·h/L. In this study, the infusion time of 37 patients was one hour, and that of others was half an hour or two hours. To ensure the accuracy of the results, only 37 patients contributed 275 plasma samples were enrolled in LSS analysis.

**Figure 4:**
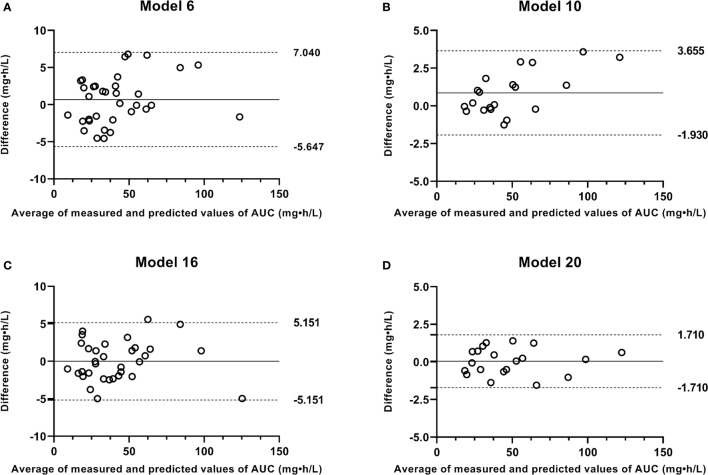
Bland-Altman plots of measured AUC versus predicted AUC values by Bayesian approach and limited sampling strategy. **(A)** model 6; **(B)** model 10; **(C)** model 16; **(D)** model 20.

Ten best-performing strategies using the Bayesian approach were displayed in **Table 4**. The steady-state trough concentration (C_0h_) and peak concentration (C_1h_) showed a medium correlation with AUC_0-12h_ (r^2^ = 0.862 and 0.846). While C_2h_ and C_4h_ both had a good correlation with AUC_0-12h_ (r^2^ = 0.922 and 0.976). For the two, three, and four-point samples, all models showed a good agreement with the AUC_0-12h_ (r^2^ ≥ 0.98). With the increase of sample points, the PE% range was narrowed down and RMSE value became small, which was consistent with the ICC and limits of agreement results. In BA analysis plots of models 6 and 10 (**Figure 4**), all points were within ±10% of the limits of agreement.

**Table 4:** The Bayesian approach of AUC_0–12h_.

Model	N	Time	r^2^	PE range (%)	RMSE	ICC (95% Cl)	Limits of agreement (%)
1	37	C_1h_	0.846	−36.45 to 54.64	22.73	0.830 (0.686–0.912)	−21.67 to 26.72
2	37	C_0h_	0.862	−41.27 to 45.76	21.85	0.914 (0.832–0.957)	−15.47 to 21.15
3	34	C_2h_	0.922	−23.98 to 47.67	18.76	0.942 (0.887–0.971)	−12.01 to 15.29
4	37	C_4h_	0.976	−14.57 to 31.33	12.80	0.985 (0.970–0.992)	−8.833 to 7.358
5	37	C_0h_+C_4h_	0.988	−15.62 to 22.78	9.34	0.994 (0.988–0.997)	−5.801 to 4.638
6	34	C_0h_+C_2h_	0.984	−16.30 to 19.23	9.81	0.991 (0.982–0.995)	−5.647 to 7.040
7	31	C_2h_+C_8h_	0.990	−15.83 to 11.84	7.73	0.987 (0.934–0.996)	−3.443 to 8.322
8	33	C_1h_+C_4h_+C_8h_	0.996	−10.48 to 6.36	4.68	0.996 (0.982–0.998)	−2.338 to 4.976
9	20	C_1.5h_+C_4h_+C_8h_	0.998	−8.94 to 3.41	4.42	0.997 (0.971–0.999)	−1.763 to 5.154
10	20	C_1h_+C_1.5h_+C_4h_+C_8h_	0.998	−5.46 to 2.85	3.26	0.998 (0.987–0.999)	−1.930 to 3.655

AUC_0–12__h_, the area under the concentration-time curve from 0 h to 12 h; PE, prediction error; RMSE, root mean square error; ICC, intraclass correlation coefficient.

The simple and multiple regression equations for estimating AUC_0-12h_ with good correlation r^2^ were shown in **Table 5**. C_2h_ model and all models using two to four samples met the acceptance criteria (Van den Elsen et al., 2018; Zhang et al., 2018). With the increase of sample points, the linear correlations between predicted and measured AUC_0-12h_ were improved. In BA analysis plots of models 16 and 20 (**Figure 4**), all points were within ±10% of the limits of agreement. Based on the above analysis, all two, three, and four-point models met the criteria for acceptability in the clinic, and the four-point strategy was the best LSS.

**Table 5:** The linear regression analysis of AUC_0–12h_.

Model	Time	Equation	r^2^	PE range (%)	RMSE	ICC (95% Cl)	Limits of agreement (%)
11	C_1h_	Y=1.345+5.338×C_1h_	0.747	−36.59 to 104.66	32.15	0.858 (0.742–0.925)	−23.74 to 23.74
12	C_0h_	Y=14.009+14.958×C_0h_	0.803	−41.95 to 79.78	27.13	0.894 (0.803–0.944)	−19.39 to 21.02
13	C_2h_	Y = −2.099+8.763×C_2h_	0.937	−34.17 to 36.28	16.33	0.968 (0.938–0.984)	−12.22 to 12.22
14	C_4h_	Y = 0.533 + 9.876×C_4h_	0.962	−29.91 to 33.13	13.07	0.981 (0.964–0.990)	−9.196 to 9.196
15	C_0h_+C_4h_	Y = 1.608 + 4.574×C_0h_+7.602×C_4h_	0.986	−26.22 to 19.60	9.07	0.993 (0.987–0.996)	−5.558 to 5.558
16	C_0h_+C_2h_	Y = −0.673+6.048×C_0h_+6.230×C_2h_	0.989	−19.18 to 18.80	8.50	0.995 (0.989–0.997)	−5.151 to 5.151
17	C_2h_+C_8h_	Y = −0.274+4.761×C_2h_+7.181×C_8h_	0.992	−9.28 to 23.93	6.67	0.996 (0.992–0.998)	−4.405 to 4.405
18	C_1h_+C_4h_+C_8h_	Y = 0.523 + 0.882×C_1h_+4.697×C_4h_+ 6.099×C_8h_	0.997	−8.08 to 16.68	4.43	0.999 (0.997–0.999)	−2.628 to 2.628
19	C_1.5h_+C_4h_+C_8h_	Y = 0.599 + 1.964×C_1.5h_+3.169×C_4h_+6.633×C_8h_	0.998	−7.66 to 5.70	3.29	0.999 (0.998–1.0)	−2.295 to 2.295
20	C_1h_+C_1.5h_+C_4h_+C_8h_	Y = 0.260 + 0.460×C_1h_+1.137×C_1.5h_+3.644×C_4h_+6.480×C_8h_	0.999	−3.83 to 4.33	2.40	1.0 (0.999–1.0)	−1.710 to 1.710

AUC_0–12__h_, the area under the concentration-time curve from 0 h to 12 h; PE, prediction error; RMSE, root mean square error; ICC, intraclass correlation coefficient.

## Discussion

To date, few studies have been conducted on population PK of polymyxin B (Kwa et al., 2008; Sandri et al., 2013; Kubin et al., 2018; Manchandani et al., 2018; Miglis et al., 2018). To our best knowledge, this study included the largest samples and most covariates from acutely-ill patients, aimed at evaluating the population PK of polymyxin B and identifying potential factors influencing the PK variability. The result indicated a two-compartment model adequately described population PK of polymyxin B in Chinese patients. Although there were studies performed using the one-compartment model (Kwa et al., 2008; Kubin et al., 2018; Manchandani et al., 2018), which may be due to limited blood points collected at distribution and elimination phases. This study showed that the median polymyxin B CL was 1.786 L/h, which was less than those reported previously (range, 1.87–2.5 L/h). For V estimate, the median was 6.218 L, closed to 6.35 L reported by Sandri et al. (2013), and was quite different from 20.39 to 33.77 L reported by Avedissian et al. (2018) and Thamlikitkul et al. (2017). With regard to V2 and Q estimates, the values were variant in different literature, probably because variability in population PK parameter estimates was high, with CV% often >30% (20.6–73.3%) (Sandri et al., 2013; Avedissian et al., 2018; Miglis et al., 2018; Avedissian et al., 2019).

The impact of clinical variables on population PK models of polymyxin B was always inconsistent, especially the influence of body weight and CrCL on polymyxin B clearance. FDA package inserts suggested polymyxin B dose reduction in patients with renal insufficiency, which was inconsistent with the recommendation provided by international consensus guidelines for the optimal use of polymyxins (Li et al., 2019; Tsuji et al., 2019). With a low correlation coefficient (**Supplementary Figure 1**), this study found CrCL was a statistical covariate of polymyxin B clearance (Equ. 6), which was in agreement with other studies (Avedissian et al., 2018; Manchandani et al., 2018). However, Manchandani et al. (2018) indicated the effect was clinically insignificant. Furthermore, by comparing the clearance and exposure of polymyxin B in normal renal function patients (CrCL ≥ 80 ml/min) and renal insufficient patients (CrCL < 80 ml/min), Thamlikitkul et al. (2017) reported the exposure of polymyxin B was similar between two groups (*P* = 0.8) after standardizing AUC for the daily dose. The clearance value of renal insufficient group was lower than that of normal function group (2.0 L/h vs. 2.5 L/h, *P* = 0.06), but there was no statistical difference. This might be because renal clearance of polymyxin B was only a minor elimination pathway in both critically ill patients and animals (Zavascki et al., 2008; Abdelraouf et al., 2012; Manchandani et al., 2016b). Taken together, given the limited cases, a small number of samples collected from each patient, and different compartment models, most of the studies, including ours, found a borderline effect of CrCL on polymyxin B clearance, the correlation needed further investigation.

As for body weight, no correlation between total body weight and clearance was observed in this study. This might be because the body weight range (45–98 kg) was narrow. With a wide range of body weight (41–250 kg), Sandri et al. (2013) demonstrated that total body weight influenced the PK parameters of polymyxin B and total body clearance, and suggested loading and maintenance doses of polymyxin B were best scaled by total body weight. Further research reported the adjusted body weight rather than the total body weight may be a better factor in influencing polymyxin B exposure (Miglis et al., 2018).

Based on the 50^th^ percentile of simulated AUC_24h_, after administration of three dosage regimens, the AUC_24h_ for patients with normal renal function and renal hyperfunction was ranged from 37.92 to 108.57 mg·h/L, which was quite different from patients with renal insufficiency (87.20–167.90 mg·h/L). Accordingly, for patients with normal renal function and renal hyperfunction, a regime of 75 to 100 mg maintenance dose twice daily would be sufficient to reach the therapeutic window (AUC_ss,24_ _hr_ of 50–100 mgċhour/L; Tsuji et al., 2019). While, as for patients with renal dysfunction, the regime of 50 mg maintenance dose twice daily would be a better option. These observations were in agreement with a previous study, although it did not provide a dosing recommendation for patients with renal dysfunction by using a one-compartment model (Manchandani et al., 2018).

Since obtaining a full concentration-time curve to calculate the AUC is not always feasible in the clinic, LSS offers a practical approach to estimating the AUC. As shown in **Tables 4** and **5**, the results of single-time point model by Bayesian method were more accurate than that of linear regression analysis, and the results of multiple-time point models obtained by the two methods were similar. In single time-point models, C_4h_ showed a better ability to predict polymyxin B AUC than C_0h_, C_1h_, and C_2h_ in both methods. It was likely that C_0h_, C_1h_, and C_2h_ were obtained in elimination, absorption, and distribution phases; while, C_4h_ was obtained between distribution and elimination phases, which could better predict polymyxin B exposure. In multiple time-point models, all models (**Tables 4** and **5**) displayed a good agreement with the measured AUC_0-12__h_ (r^2^ ≥ 0.98), which was acceptable for polymyxin B TDM. The 4-point models (C_1h_, C_1.5h_, C_4h_, and C_8h_), which included the absorption, distribution, and elimination phases, were the best predictor of polymyxin B AUC_0-12h_. Additionally, it was reported a 2-point model including C_0h_, and C_2h_ was appropriate for colistin TDM (r^2^ = 0.98) because the C_0h_ sample had an association with renal toxicity and the C_2h_ sample was essential to monitor efficacy (Kim et al., 2019). This result was in agreement with that of model 16 in this study. Accordingly, models 5 to 10 and 15 to 20 all can be recommended for polymyxin B TDM, especially C_0h_-C_2h_ and C_1h_-C_1.5h_-C_4h_-C_8h_ models.

Our study has several limitations. Firstly, this study enrolled a relatively small number of patients, leading to a lack of external validation of the population PK model and LSS. Future studies should evaluate the PK of polymyxin B with a larger population. Second, since patients with diverse underlying conditions, drug-drug interactions, and co-administration of other antibiotics were not included in the population PK model. Third, individual PK parameters may change during therapy occasions. Through the modeling, ignoring inter-occasion variability may lead to a big bias in parameter estimates, especially for drugs with large intra-individual variability that require TDM (Karlsson and Sheiner, 1993; Abrantes et al., 2019). However, since all data were collected on day four, whether this population PK model is suitable for other occasions is still unknown. Fourth, the LSS results were only applicable to patients who intravenously infused polymyxin B for one hour. Finally, the efficacy and toxicity thresholds based on clinical outcomes were not investigated in this study, which was the next work of our group.

## Conclusion

In conclusion, a two-compartment population PK model was successfully established to characterize the PK parameters of polymyxin B in Chinese patients with MDR Gram-negative bacterial infections. Furthermore, as far as we know, this is the first study to develop and validate the LSS of polymyxin B. The results suggested 2-point model (C_0h_ and C_2h_) and 4-point model (C_1h_, C_1.5h_, C_4h_, and C_8h_) performed well in predicting polymyxin B AUC, which could be applied in clinical practice to assist TDM of polymyxin B.

## Data Availability Statement

All datasets generated for this study are included in the article/**Supplementary Material**.

## Ethics Statement

The studies involving human participants were reviewed and approved by Zhengzhou University Medical Research and Ethics Committee. The patients/participants provided their written informed consent to participate in this study.

## Author Contributions

PW and JY designed the research. QZ performed the experiments. ZZ analyzed the results. MF, TS, and XZ supervised the research and revised the manuscript. All authors approved the final manuscript.

## Funding

This work was supported by the National Natural Science Foundation of China (Grant No. 81703799 and 81803638).

## Conflict of Interest

The authors declare that the research was conducted in the absence of any commercial or financial relationships that could be construed as a potential conflict of interest.
