# Supplementary material for: Population Pharmacokinetics and Limited Sampling Strategy for Therapeutic Drug Monitoring of Polymyxin B in Chinese Patients With Multidrug-Resistant Gram-Negative Bacterial Infections
Source: Front Pharmacol. 2020 Jun 5;11:829. doi: 10.3389/fphar.2020.00829 (PMC7289991; doi:10.3389/fphar.2020.00829)
Supplement: Supplementary file 1 [file Image_1.pdf]

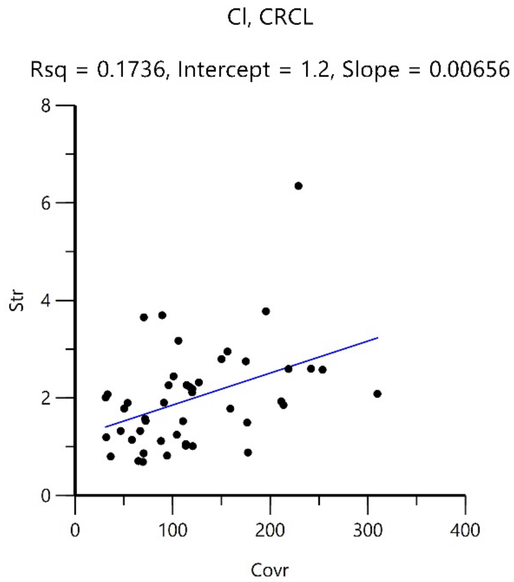

Supplementary Figure 1. The covariate plots of creatinine clearance (CrCL) versus central compartment clearance (Cl). Covr, covariates (CrCL, mL/min); Str, individual model estimated structural parameter values (Cl, L/h).
